# Dissociable Neural Systems for Timing: Evidence from Subjects with Basal Ganglia Lesions

**DOI:** 10.1371/journal.pone.0010324

**Published:** 2010-04-23

**Authors:** H. Branch Coslett, Martin Wiener, Anjan Chatterjee

**Affiliations:** Department of Neurology, School of Medicine, University of Pennsylvania, Philadelphia, Pennsylvania, United States of America; The Research Center of Neurobiology-Neurophysiology of Marseille, France

## Abstract

**Background:**

The neural basis of timing remains poorly understood. Although controversy persists, many lines of evidence, including studies in animals, functional imaging studies in humans and lesion studies in humans and animals suggest that the basal ganglia are important for temporal processing [1].

**Methodology/Principal Findings:**

We report data from a wide range of timing tasks from two subjects with disabling neurologic deficits caused by bilateral lesions of the basal ganglia. Both subjects perform well on tasks assessing time estimation, reproduction and production tasks. Additionally, one subject performed normally on psychophysical tasks requiring the comparison of time intervals ranging from milliseconds to seconds; the second subject performed abnormally on the psychophysical task with a 300ms standard but did well with 600ms, 2000ms and 8000ms standards. Both subjects performed poorly on an isochronous rhythm production task on which they are required to maintain rhythmic tapping.

**Conclusions/Significance:**

As studies of subjects with brain lesions permit strong inferences regarding the necessity of brain structures, these data demonstrate that the basal ganglia are not crucial for many sub- or supra-second timing operations in humans but are needed for the timing procedures that underlie the production of movements. This dissociation suggests that distinct and dissociable processes may be employed to measure time intervals. Inconsistencies in findings regarding the neural basis of timing may reflect the availability of multiple temporal processing routines that are flexibly implemented in response to task demands.

## Introduction

Space and time are widely considered to be the elementary dimensions of human experience. Although substantial progress has been made in understanding spatial processing, the neural basis of temporal processing remains poorly understood. A number of lines of evidence suggest that the basal ganglia are crucial for timing [1]. For example, electrophysiologic studies in animals have demonstrated patterns of neuronal firing in the basal ganglia that appear to encode the duration of stimulus events. Matell, Meck and Nicolelis [2] demonstrated that neurons in the dorsal-anterior striatum of rats ‘peak’ in their firing rates at the same time as maximal lever pressing during a timing task; both patterns coincide with the criterion duration. Chiba, Osio and Inase [3] recorded from monkey striatal neurons during a temporal discrimination task; consistent with the claim that the striatum is a component of a clock mechanism, they demonstrated that different populations of striatal neurons phasically altered their firing rate depending on the interval of the presented stimuli.

A number of neuroimaging studies demonstrate basal ganglia activation during temporal processing tasks (for review see [4]). For example, Rao, Mayer and Harrington [5] demonstrated that basal ganglia activation was restricted to the encoding of interval duration, rather than comparison processes, while Bueti et al. [6] demonstrated that the basal ganglia were active during timing tasks whether a timed motor response was required or not. Studies of rhythmic tapping behavior demonstrate that the basal ganglia exhibit greater activation when the subject is required to tap without external pacing [7],[8].

Studies of subjects with brain dysfunction have also been taken as evidence in support of the role of the basal ganglia in timing. In some studies, subjects with Parkinson's Disease have demonstrated deficits on a variety of timing tasks [9],[10]. Other studies involving subjects with Parkinson's Disease, however, have not demonstrated deficits on timing tasks such as rhythmic finger tapping [11], [12] or, in the report of Wearden et al [13], a variety of measures of timing that do not involve a motor response. A number of studies have demonstrated that Huntington's Disease, a disorder that causes a degeneration of the neostriatum, is associated with substantial impairments in timing [14]; this abnormality is observed even in subjects with this disorder who are in many other respects pre-symptomatic (e.g., [15]). Alterations of temporal processing have also been reported in disorders such as ADHD [16]; [17], Tourette's Syndrome [18] and schizophrenia [19] in which altered dopaminergic transmission has been implicated.

Studies involving subjects with focal lesions of the basal ganglia have been less definitive. Whereas bilateral lesions of the basal ganglia in rats lead to gross timing impairments [20], studies of humans with unilateral lesions have failed to find a deficit [21]. We are unaware of any investigations of timing in human subjects with bilateral basal ganglia focal lesions. This is important because many patients with focal, unilateral basal ganglia lesions have little or no clinical evidence of basal ganglia dysfunction; the absence of deficits on timing tasks in these subjects might, therefore, reflect compensation by intact structures in the contralateral hemisphere or the fact that the impact of the lesion was modest.

We report data from two subjects with extensive neuroimaging documented bilateral basal ganglia lesions and clinical evidence of basal ganglia dysfunction. Experimental tasks were designed to interrogate a number of different timing operations with both sub- and supra-second stimuli. We note that tasks were not selected on the basis of theoretical considerations but were chosen to be easily understood and reliably executed by our aging subjects. Both subjects perform well on a variety of tasks employing sub- and supra-second stimuli, but are significantly impaired on tasks requiring rhythmic timing. These data suggest that multiple procedures may be employed for interval timing and that the basal ganglia are crucial only for the timing procedures underlying the production of rhythmic movements.

## Materials and Methods

### Ethics Statement

The investigations were approved by the Institutional Review Board at the University of Pennsylvania. The work was conducted according to the principles expressed in the Declaration of Helsinki. Written informed consent was obtained from all participants.

### Subjects

**Figure 1:**
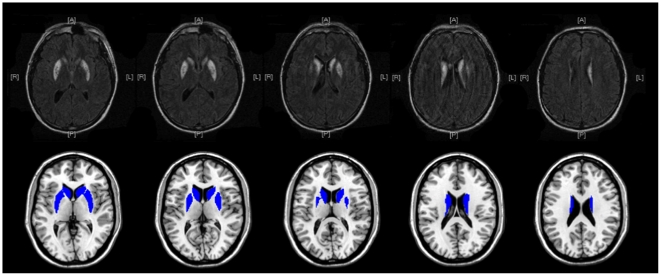
FLAIR MRI images from XG demonstrating injury to the caudate, putamen and globus pallidus; white regions indicate areas of damage. Overlay images of the intact basal ganglia on a template brain are displayed for reference purposes.

Subject one (XG) is a right-handed 48 year-old disabled tradesman who suffered hypoxic encephalopathy as a consequence of cardiac arrhythmia resulting in disabling motor deficits eight years prior to the testing reported here. Examination demonstrated prominent signs of basal ganglia dysfunction including dystonic posturing, rigidity and akinesia. He performed well on a general cognitive screening test; there was no evidence of amnesia, aphasia or attentional impairment. He had cortical blindness during his initial hospitalization, which resolved completely in a few months. MRI scan demonstrated extensive lesions of the caudate, putamen and globus pallidus bilaterally (Figure 1). Although the data must be interpreted with caution as the measure was developed specifically for subjects with Parkinson's Disease, data from the motor exam of the Unified Parkinson's Disease Rating Scale (UPDRS) for both subjects are provided in Table 1.

**Table 1:** Motor Exam of the Unified Parkinson's Disease Rating Scale.

	XG	KQ-167
Speech	1	2
Facial Expression	2	3
Tremor at Rest	0	0
Action or Postural Tremor	0	0
Rigidity		
Neck	1	2
Right Arm	2	3
Left Arm	3	2
Right Leg	2	3
Left Leg	2	3
Finger Taps		
Right	2	2
Left	2	2
Hand Movements		
Right	2	2
Left	3	2
Rapid Alternating Movements		
Right	1	2
Left	2	2
Leg Agility		
Right	1	3
Left	1	3
Arising from Chair	2	4
Posture	0	2
Gait	1	3
Postural Stability	1	3
Body Bradykinesia/Hypokinesia	2	3

**Figure 2:**
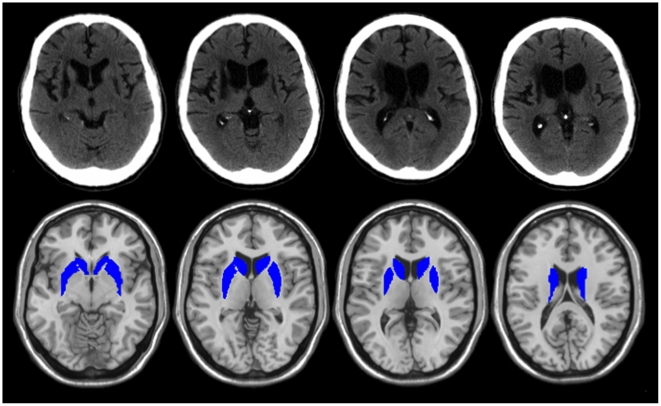
CT images from KQ-167 demonstrating loss of the caudates bilaterally (with ventricular enlargement) and infarcts in the right and left lenticular nuclei. Overlay images of the intact basal ganglia on a template brain are displayed for reference purposes.

Subject two (KQ-167) is a right-handed 54 year-old disabled laborer who suffered a stroke involving the right basal ganglia and a stroke involving the left basal ganglia nine and seven years, respectively, prior to the testing reported here. Examination demonstrated him to be wheel-chair confined and to exhibit clinical signs of basal ganglia impairment including dystonic posturing, akinesia and bradykinesia; no tremor was noted. He performed well on a general cognitive screening test; there was no evidence of aphasia, attentional impairment or visuo-spatial deficit. MRI scan demonstrated extensive lesions of the caudate, putamen and globus pallidus bilaterally (Figure 2). Thirteen right-handed age-matched controls (mean age 52±8 years) with no history of neurologic or psychiatric disease participated in experiment 1. Ten subjects from this group participated in experiments 2 and 3.

### Experiment 1: Estimation, Reproduction and Production of Supra-second Intervals

A battery of tasks assessing temporal estimation, production and reproduction at the supra-second level was administered. Interval estimation was assessed by asking subjects to indicate the duration of a visual or auditory stimulus. At the onset of each trial, a central fixation point consisting of a filled black circle (0.5 cm diameter) was presented in the middle of the screen for one second, after which a stimulus was presented for 2, 4, 6, 8, 10 or 12 seconds. In the auditory version of the task, a free-field 250 Hz tone adjusted to a comfortable volume level was presented, whereas in the visual version of the task, a 4×4 cm red square was presented in the middle of the computer screen. At the offset of the stimulus, subjects were prompted by the word “respond” to indicate, in seconds, how long they believed the stimulus was present; subjects were told to respond with whatever precision they desired (that is, seconds, tenths of a second, etc.). For this and all other tasks in Experiment 1, stimuli for each of the six durations were presented five times in random sequence for each modality. Subjects were not told the range of stimulus durations and were not given feedback regarding accuracy.

Interval production was assessed by asking subjects to generate an interval of a designated duration. At the onset of each trial, a fixation point was presented in the middle of the screen for one second. The fixation point was replaced by a number (2, 4, 6, 8, 10, or 12) that indicated the duration of the interval to be generated. The subjects initiated the stimulus onset by depressing the space bar on the keyboard. When the subjects believed the required interval had elapsed, they pressed the space bar a second time to terminate the trial. In the auditory version of the task, depressing the space bar generated the same tone used in the duration estimation task; in the visual task, depressing the space bar generated the same red square used in the estimation task.

Interval reproduction was assessed by asking subjects to observe and then reproduce a visual stimulus. At the onset of each trial, a fixation point was presented in the middle of the screen for one second. Following this, the fixation point extinguished and was replaced by a red square. The stimulus persisted for a fixed duration (2, 4, 6, 8, 10, or 12 seconds). After the prescribed duration, the stimulus extinguished and subjects initiated the reproduction stimulus by pressing the space bar causing the red square to appear; subjects pressed the space bar a second time when they believed the target interval had been reached. All three tasks were administered in the order they are reported (auditory estimation, visual estimation, auditory production, visual production, visual reproduction). XG and KQ-167 performed the above tasks twice, on separate days; during the second session, the tasks were run in the reverse order.

For Experiments 1 and 2, subjects were asked not to use a counting strategy. No counting, tapping, nodding or other repetitive movements were observed. All tasks were performed with a laptop computer with a 38 cm screen and a refresh rate of 60 Hz. All subjects sat at a desk with the computer screen at a distance of approximately 50 cms. An experimenter was present during all experimental trials and monitored the subject to ensure attention was focused on the present task.

#### Data Analysis

The mean response time for each task (estimation, production, and reproduction) was plotted against the stimulus interval. Comparisons between patient and control scores were carried out separately for XG and KQ-167. The mean response times for each subject in each task were fit with a linear regression (y = y_0_ + ax) and slope values were obtained; the slope values were tested for differences between individual patients and controls. As a measure of variability, we utilized the coefficient of variation (CV; standard deviation/mean response time); the CV for each duration was tested for differences between the individual patient CV and the average normal control CV. For all single-score comparisons between patient and controls we utilized the Crawford & Howell modified one-tailed *t*-test for significant differences in single-case studies [22] expressed in the following formula:
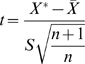
Where X^*^ is the patient score, 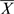 is the control average, and *S* is the standard deviation of control scores. Significance level was always set to α = 0.05.

### Experiment 2: Temporal Discrimination at Sub- and Supra-second intervals

This task was designed to assess non-motor aspects of temporal perception across the sub-second and supra-second range with a temporal discrimination task in which subjects were asked to judge which of two intervals was longer. The Parameter Estimation by Sequential Testing (PEST) algorithm [23] was used to estimate temporal discriminability for target intervals of 300, 600, 2000, and 8000 milliseconds. The PEST algorithm is an adaptive staircase procedure that uses a subject's responses to derive a probability-based estimate based on a normal sigmoid-shaped psychophysical function to generate a different comparison interval on each successive trial. Each target interval was tested in random order, in separate blocks consisting of 60 trials. Prior to testing, subjects received 30 practice trials with a standard duration of 1000ms. For each trial, a fixation point identical to that used in Experiment 1 was presented for one second, followed by a 4×4 cm red square for one of the above target intervals (standard duration); after an interval of one second during which the screen was blank, a second red square was then presented for a variable duration of time (comparison duration) as determined by the adaptive staircase procedure of the PEST algorithm. The comparison duration boundaries were initially set to 150% or 50% of the standard interval for determining upper and lower thresholds respectively. For example, on trials with a 600ms standard, the shortest initial comparison duration would be 300ms and the longest comparison would be 900ms. For the first 20 trials, the comparison interval was adjusted up 15% steps of the standard interval; all successive comparison intervals were adjusted in 5% steps. As a consequence of the design, each subject experienced a different set of comparison durations. Subjects pressed the “L” key if they judged the second stimulus to be longer or the “S” key if they judged the second stimulus to be shorter. Subjects were not told the range of stimulus durations and were not given feedback regarding accuracy.

#### Data Analysis

For each of the four standard intervals (300 ms, 600 ms, 2000 ms, 8000 ms), the probability of the subject making a “longer” response choice was plotted as a function of the comparison interval. This data was then fit with a sigmoidal, psychometric curve using the psignifit version 2.5.6 software package (see http://bootstrap-software.org/psignifit/) for Matlab, which implements the maximum-likelihood method described by Wichmann & Hill [24]. Upper and lower thresholds, the approximate points at which the subject is 25% or 75% likely to judge the stimulus as longer, were calculated using the bias corrected (BC) bootstrap method implemented by psignifit, based on 4999 simulations [25]. The results of this analysis yield the point of subjective equality (PSE; the time value when subjects were equally likely to judge the stimulus as longer or shorter), the difference limen (DL; [upper – lower thresholds]/2), and the coefficient of variation (CV; difference limen/PSE). Each value was averaged across normal controls and compared to the patient's scores.

### Experiment 3: Timed Tapping

A timed, repetitive tapping task was also administered. After initiating a trial with a key press, subjects were presented with an isochronous series of 440 Hz tones; the tones were 50ms in duration and were presented at 400 ms intervals. Subjects were instructed to observe the stimuli until they felt comfortable that they understood the pattern of occurrence, and then begin tapping a response key in time with the stimuli. Stimuli were presented until 14 taps were recorded (synchronization phase), after which the auditory stimulus was extinguished and subjects continued to tap at the same rate (continuation phase) for 31 taps. Feedback in the form of a normalized average response time (mean inter-tap-interval (ITI) divided by 400) was provided after each block. Subjects responded with the each hand separately for 12 blocks of taps with 24 blocks of trials per session; blocks with the right and left hand were randomly interspersed.

#### Data Analysis

Only tapping from the continuation phase was analyzed, and the first tap from each trial was removed from the analysis. The original analysis for this experiment utilized the two-process model developed by Wing & Kristofferson [26] for parsing the observed ITI variance into discrete components. In this analysis, drift was accounted for by fitting the tap times within each trial with a regression line; the residuals from each regression were then used to calculate the lag 1 autocovariance in order to further calculate central and motor variance scores (for a further discussion of methods and theory see [27]). As encountered by other investigators [28], [29], we found violations of the Wing & Kristofferson model across all subjects. The violations took the form of positive autocovariance values. Furthermore, both patients exhibited significantly more violations than control subjects (XG violations: 9, (t) = 2.492, *p* = 0.017; KQ-167 violations: 10, (t) = 2.972, *p* = 0.007; Mean control violations: 3.8±1.9). Although different methods have been proposed to address these violations [30], the fact that patient violations significantly exceeded controls suggested that the model would be unreliable in documenting differences between patients and controls. Consequently, we restricted our analysis to the average ITI and CV of tapping responses. Mean ITI and variability (CV) scores were separately compared against controls for both XG and KQ-167.

## Results

### Experiment 1

**Figure 3:**
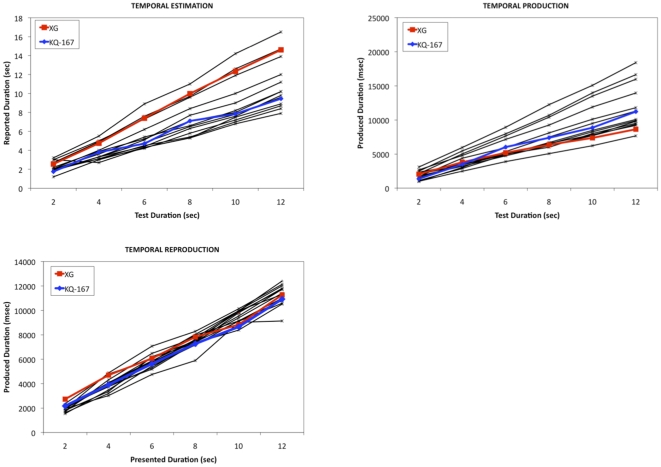
Data from a temporal estimation, production and reproduction task for both individual subjects and controls for time intervals 2–12 seconds.

**Figure 4:**
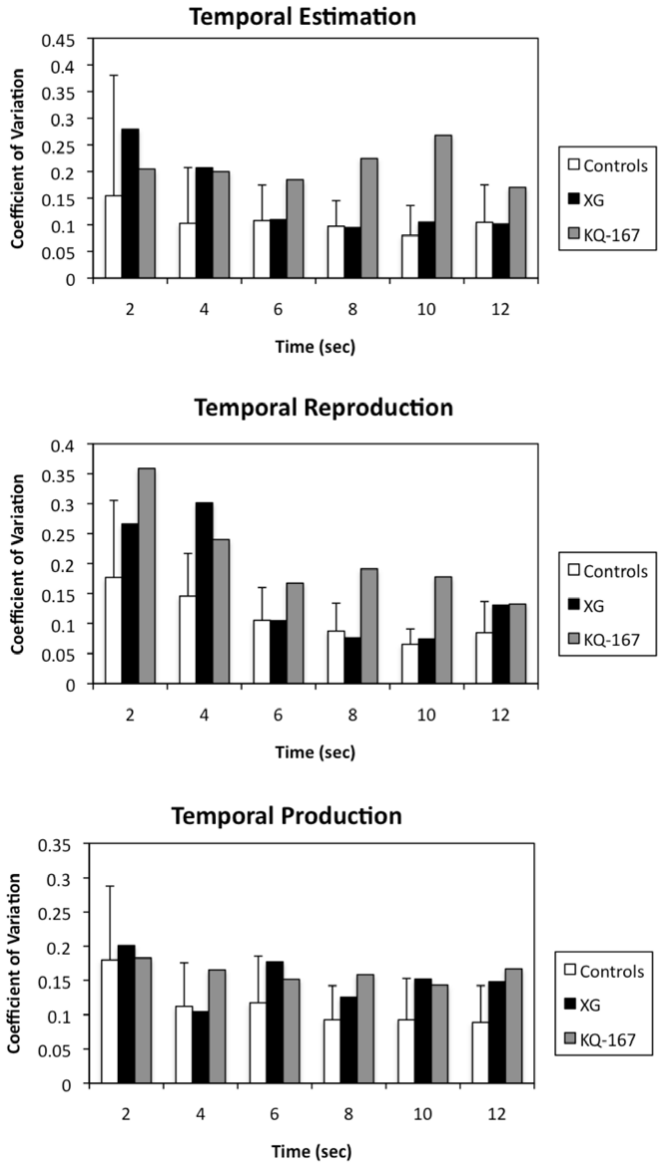
CV scores at each duration for temporal estimation, production and reproduction tasks for both individual subjects and controls.

For both XG and KQ-167, as performance did not differ in the two administrations of the task the data were combined. Linear regressions for both patients and controls fit the data equally well (Estimation R^2^: Controls = 0.99±0.01, XG = 0.99, KQ-167 = 0.98; Production R^2^: Controls = 0.99±0.003, XG = 0.99, KQ-167 = 0.99; Reproduction R^2^: Controls = 0.99±0.009, XG = 0.99, KQ-167 = 0.99; see Table S1). As demonstrated in Figures 3 and 4, XG and KQ-167 performed normally with respect to accuracy, as defined by the slope, and variability, as defined by the CV, for the reproduction, production and estimation tasks (all *p*>0.05)

### Experiment 2

As shown in Table 2, XG performed normally at all four standard intervals with respect to accuracy (PSE) and variability (DL and CV). KQ-167 performed normally with 600, 2000 and 8000 ms. stimuli but exhibited a significant prolongation at 300 ms. with a PSE of 529 ms. [t(9) = 3.056, *p* = 0.006]; his difference threshold and coefficient of variation were normal at all four intervals (all *p*>0.05).

**Table 2:** Temporal discrimination data for XG, KQ-167 and controls (with between-subject standard deviations).

PSE	300	600	2000	8000
Controls	0.33 (±0.06)	0.62 (±0.04)	1.92 (±0.22)	7.74 (±0.49)
XG	0.29	0.55	1.58	7.29
KQ-167	0.53*	0.69	1.78	7.44

DL	300	600	2000	8000
Controls	0.12 (±0.06)	0.17 (±0.05)	0.44 (±0.17)	1.61 (±0.54)
XG	0.09	0.14	0.34	0.87
KQ-167	0.05	0.12	0.42	1.09

CV	300	600	2000	8000
Controls	0.35 (±0.12)	0.27 (±0.08)	0.22 (±0.08)	0.25 (±0.12)
XG	0.31	0.25	0.21	0.12
KQ-167	0.16	0.20	0.21	0.14

Data are displayed for point of subjective equality (PSE), difference limen (DL) and coefficient of variation (CV) scores for four possible standard durations. Asterisks represent p<0.05.

### Experiment 3

**Figure 5:**
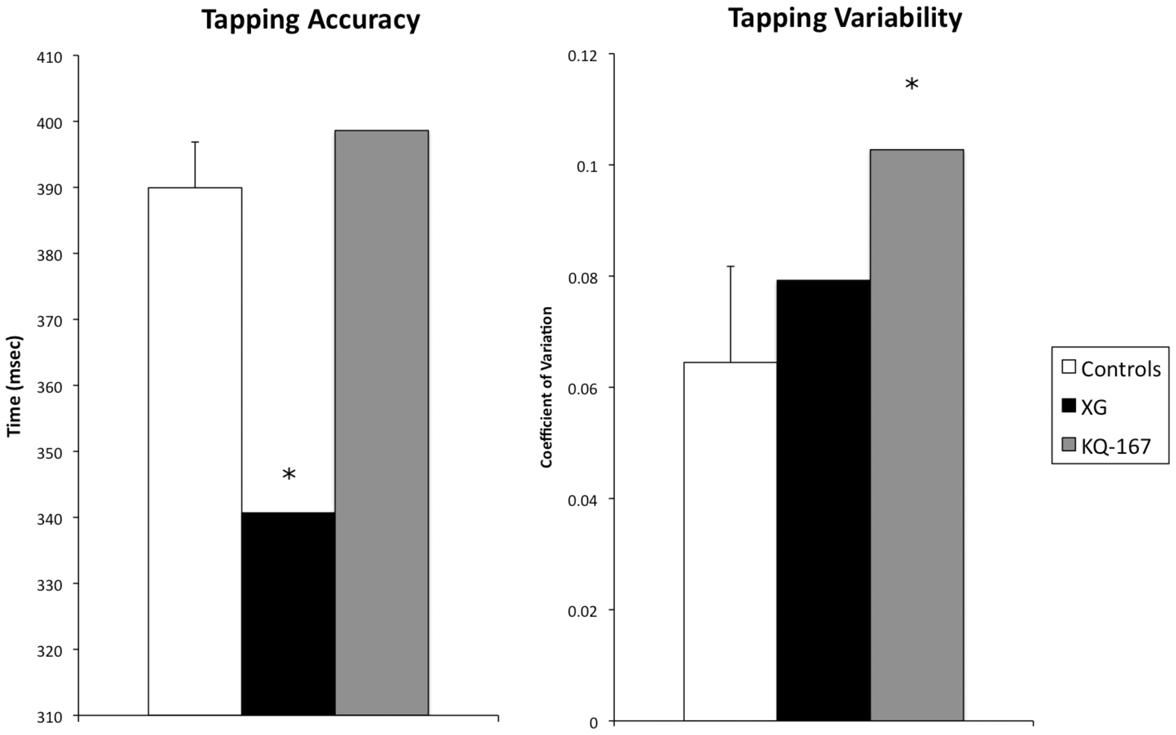
Data from the tapping task demonstrating increased abnormal accuracy for XG and increased variability for KQ-167. Error bars for control subjects represent between-subject standard error, whereas error bars for patients represent between-trial standard error. Asterisks represent p<0.05.

As indicated in Figure 5, XG performed abnormally with respect to accuracy [t(9) = −2.018, *p* = 0.037], producing abnormally short intervals. XG demonstrated normal variability in this range [t(9)] = 0.814, *p* = 0.218]. KQ-167 exhibited normal accuracy [t(9) = 0.377, *p* = 0.357] but significantly elevated variability [t(9) = 2.110, *p* = 0.032].

## Discussion

Despite clinical and radiologic evidence of substantial bilateral basal ganglia disruption, XG and KQ-167 perform well on a wide range of timing tasks including estimation, production, reproduction and discrimination with both sub- and supra-second stimuli. These data support several important conclusions. First, the findings represent a challenge to the view that the basal ganglia are crucial for timing [9], [31], [1] as well as a leading account of interval timing, the Striatal Beat Frequency model [32]. On the latter account, multiple cortical regions contain neurons that exhibit properties (e.g., oscillations at different rates, ramping behaviour) that would be appropriate for measuring time intervals; these neurons are assumed to project to the medium spiny neurons of the striatum where they are integrated to detect patterns of input that correspond to a specific time interval (see [32]). As recently noted by Meck and colleagues [33], [1], substantial pharmacologic data in humans (see [31]) as well as electrophysiologic studies in animals [34] and some imaging studies in humans support this account [1]. The demonstration that two subjects with extensive and disabling focal lesions of the bilateral basal ganglia perform normally on a wide range of timing tasks with sub- and supra-second stimuli suggest that the basal ganglia are not crucial for many interval timing procedures.

Our findings are at odds with a number of previous investigations of subjects with basal ganglia dysfunction. A substantial body of literature has demonstrated that subjects with degenerative diseases of the basal ganglia, such as Parkinson's or Huntington's Disease, exhibit significant impairments in interval timing [9], [29], [10], [14], [35], [15]; but see [13]. Harrington et al [9], for example, reported data from 24 subjects with Parkinson's Disease who were impaired on duration perception and finger tapping tasks. Similarly, Malapani and colleagues [10] have demonstrated that subjects with Parkinson's Disease exhibit a “migration effect” such that when asked to reproduce intervals of differing duration (e.g., 8 and 21 seconds), their responses tend to converge toward an intermediate value; this effect is reduced by L-dopa treatment.

While of interest in their own right, we suggest that investigations of patients with degenerative diseases of the brain do not permit strong brain-behavior inferences. Neurodegenerative diseases are characterized by deficits in multiple neural elements; for example, pathology in Parkinson's Disease is evident in the basal ganglia, substantia nigra, thalamus, subthalamic nucleus, dorsolateral prefrontal cortex, SMA, and elements of the peripheral nervous system [36]. In light of this widespread pathology, we believe that our data are not inconsistent with demonstrations of impairment in timing procedures in subjects with Parkinson's and Huntington's Diseases as the deficits in the latter conditions may reflect the effects of dysfunction of brain circuits other than the basal ganglia (cf., [37], [21]).

In a similar vein, the extensive literature demonstrating that pharmacologic manipulations of the dopamine system are associated with alterations in interval timing [38], [31] is not decisive with respect to the role of the basal ganglia in timing. Dopaminergic neurons project not only to the striatum but also the limbic system (mesolimbic projections) as well as the cortex (mesocortical projections), raising the possibility that manipulations of the dopaminergic system induce alternations in timing by virtue of effects at extra-striatal sites. Lewis and Miall [38], for example, emphasize the role of the mesocortical dopaminergic projections in time processing. Consistent with this view, Rammsayer [39] demonstrated that remoxipride, a drug that primarily blocks D2 receptors in the cortex, interferes with interval processing with stimuli in the seconds range whereas haloperidol, which blocks D2 receptors in both mesocortical and mesolimbic systems, disrupts interval timing for both sub- and supra-second stimuli. Thus, although there is compelling evidence that dopaminergic systems are implicated in at least some aspects of interval timing, we suggest that the pharmacologic studies do not unambiguously implicate the basal ganglia in interval timing. Data from our subjects are consistent with other accounts (e.g, [38]) that argue for a prominent role of mesolimbic and mesocortical dopaminergic projections in interval timing.

Our data demonstrating that both subjects were impaired on the timed tapping task are inconsistent with two studies in which subjects with unilateral basal ganglia focal lesions were reported (Shin et al, 2005; Aparicio et al, 2004). One potential explanation for this discrepancy is that the lesions of our subjects were bilateral whereas the lesions in the subjects reported by these investigators were relatively small and unilateral. Consistent with the differences in number and extent of the lesions, the subjects reported by Shin et al [37] and Aparicio et al [21] appear to have had little clinical evidence of basal ganglia dysfunction. Aparicio et al [21], for example, noted that their subjects exhibited minimal deficits on a variety of tasks assessing basal ganglia function.

We believe that our subjects provide a stronger test of the role of the basal ganglia in timing for several reasons. First, unlike previous reports of subjects with focal brain lesions, our subjects exhibited bilateral, severe lesions of the basal ganglia as documented by neuroimaging. Second, unlike many previously reported subjects, our subjects exhibited significant, disabling clinical signs of basal ganglia dysfunction. Thus, our subjects' good performance on many timing tasks in the context of severe basal ganglia dysfunction cannot be attributed to the fact that the lesions were insufficient to impair basal ganglia functions and suggest that negative findings from subjects with unilateral basal ganglia lesions causing minimal basal ganglia deficits should be interpreted with caution.

The discrepancy between the subjects' impaired performance on the timed tapping task as compared to the other tasks described above is consistent with the claim that timing may be mediated by distinct and dissociable routines (cf. [31] [38]). Lewis and Miall [4] identified three parameters according to which timing tasks may be distinguished. One dimension is interval duration. The distinction between “automatic” timing procedures that are relevant to short (e.g, less than 1 second) intervals and “cognitive” timing procedures that mediate the processing of relatively long time intervals (e.g., 1 second or longer) was first proposed over 100 years ago by Münsterberg [40] and has received considerable empirical support [33], [41], [42]; but see [43] for a dissenting view). A second parameter identified by Lewis and Miall [4] is the nature of the response. They distinguished between “motor timing” tasks for which the timing of the response itself provided the dependent measure of performance and “perceptual timing” tasks for which the time of the response was not important; on their account, reproduction, production and tapping tasks represent motor timing tasks whereas interval judgment and estimation tasks represent non-motor tasks. The fact that XG performed normally on tasks requiring judgments about stimuli ranging from 300 ms to 8000 ms and KQ-167 performed normally on tasks requiring judgments about stimuli ranging from 600 ms to 8000 ms suggest that interval duration is not a crucial determinant of the timing routines mediated by the basal ganglia. Similarly, the fact that both subjects performed well on some tasks for which the response was defined by movement (e.g., reproduction task and production tasks in Experiment 1, duration estimation task in Experiment 2), suggest that the basal ganglia are not central to tasks requiring a motor response. One possible explanation for the discrepancy between the good performance in Experiments 1 and 2 and the poor performance in Experiment 3 is that XG and KQ-167 were impaired specifically for repetitively timed movements. Support for this comes from the observation that subjects were not impaired on tasks for which the response required a single motor response but exhibited significant and substantial deficits on the timed tapping task in which the interval was defined by a motor response that initiated the interval and a second response that marked the end of the interval (as well as the beginning of the next interval). On the basis of these data we suggest that the basal ganglia are crucial for generating temporally precise motor patterns is consonant with accounts that emphasize the role of the basal ganglia in action.

There has been substantial debate regarding the roles of the basal ganglia and cerebellum in timing [41], [44], [38]). Spencer et al ([45]; see also Spencer and Ivry, [12]) reported data from subjects with cerebellar lesions who exhibited significant impairment in a discontinuous motor task. When asked to draw circles in a continuous, smooth but temporally precise manner subjects with cerebellar disease performed normally; in contrast, when required to pause between each circle, the subjects performed abnormally. The contrast between the performance of our subjects and those of the cerebellar lesions subjects reported by Spencer et al [45] raises the possibility that the cerebellum and basal ganglia may contribute to different aspects of temporal processing. More specifically, we suggest that the cerebellum may be important for discontinuous movements that require a representation of a temporal goal whereas the basal ganglia are crucial for continuous motor timing in which the temporal goal is embedded in action [46].

Several potential alternative accounts of our data should be considered. One issue concerns the potential role of counting or other procedures by which subjects produce repetitive or rhythmic overt or covert actions to mark the passage of time. Although all subjects were asked not to employ such as a strategy, we are unable to effectively monitor compliance with this request. Several factors suggest that a strategy such as counting cannot explain our subjects' relatively good performance, however. First, both subjects performed normally on interval judgments with sub-second stimuli for which counting would not be expected to help. Second, our subjects performed poorly on the sustained tapping task that would appear to be similar in many respects to a counting or covert action strategy; although speculative, we believe it that normal performance on multiple timing tasks was achieved by employing an impaired procedure for generating rhythmic signals.

Second, as both subjects experienced their neurologic insults several years prior to the testing reported here, one might speculate that they had initially exhibited deficits in timing but had recovered. In this context, “recovery” could take at least two forms. First, it is possible that, although damaged, the basal ganglia regained their functional capacities. This seems unlikely as the subjects continue to exhibit profound clinical deficits (e.g., rigidity, akinesia, dyskinesia) typically associated with basal ganglia disruption; the hypothesis that the subjects' perform normally on many timing tasks in the context of radiologic and clinical evidence of basal ganglia dysfunction requires one to postulate that the basal ganglia routines mediating interval timing fully resolved whereas the processes that underlie motor systems exhibited little or no improvement. A second form of “recovery” might also be invoked to explain our subjects' performance. For example, one might propose that the basal ganglia are integral to most timing procedures but that the subjects compensated by learning to employ different timing routines that do not rely on the basal ganglia. The possibility that our subjects were able to compensate for timing deficits caused by their basal ganglia lesions cannot be excluded. We note, for example, that in a recent meta-analysis of functional imaging data on timing, we found that the right inferior frontal gyrus and the bilateral Supplementary Motor Areas were the only regions active across all timing tasks [47]. One might speculate that these brain regions were recruited to support timing operations in our subjects. We suggest, however, that our findings undermine the strong claim that the basal ganglia are necessary for timing.

Finally, the fact that our subjects exhibited basal ganglia dysfunction from different etiologies (ischemic infarction and hypoxic encephalopathy) strengthens our claims. The fact that our subjects performed abnormally in the setting of different kinds of basal ganglia lesions provides evidence that the pattern of performance is not specific to the pathologic process but to lesion location. It must be noted in this context, however, that the performance of the two subjects was not identical. This is perhaps most evident in the timed tapping task (Experiment 3) in which XG was impaired with respect to accuracy and KQ-167 with respect to variability. Whether the discrepancy in performance demonstrated in Experiment 3 reflects an effect of severity of the deficit or a differential impact on components of the heterogeneous and functionally specialized brain structures that are collectively termed the “basal ganglia” cannot be stated with certainty.

We note that although our data inform theories concerning the basal ganglia, they reveal little information about what other neural regions may support temporal processing. Indeed, numerous other regions have been suggested to support timing functions, including – but not limited to – the cerebellum [28], right parietal lobe [48], [49], supplementary motor area [43] and the insular cortex [50]. Although reviews of the literature on timing have generally supported the basal ganglia timing hypothesis (e.g. [1]), there exists a lack of consistency concerning the necessity of other neural regions for timing. In a recent quantitative meta-analysis of the neuroimaging literature on temporal processing, Wiener, Turkeltaub and Coslett [47] demonstrated that many so-called “timing” regions showed differential probabilities of activation depending on the type of timing task employed. Of relevance to the present report, the basal ganglia were most likely to be activated during sub-second timing tasks with a heavy motor component. These data are consistent with our two patients, who show a dramatic impairment during timed tapping at 400ms, but relatively preserved performance on other task types.

In conclusion, we propose that the basal ganglia are not essential for many sub- and supra-second timing procedures. However, the basal ganglia do seem to mediate procedures that underlie the timing of rhythmic movements. These observations constrain accounts of the role of the basal ganglia in timing and suggest that different and neurally dissociable timing procedures are engaged depending on the demands of particular tasks.

## Supporting Information

Table S1 Individual scores for control subjects and patients for temporal estimation, production and reproduction tasks utilized in experiment 1. Each score represents the average response for that duration. Pearson correlation coefficients and R2 values for each subject are also displayed.
(0.10 MB DOC)
