# Supplementary material for: Dissociable Neural Systems for Timing: Evidence from Subjects with Basal Ganglia Lesions
Source: PLoS One. 2010 Apr 23;5(4):e10324. doi: 10.1371/journal.pone.0010324 (PMC2859062; doi:10.1371/journal.pone.0010324)
Supplement: Table S1 — Individual scores for control subjects and patients for temporal estimation, production and reproduction tasks utilized in experiment 1. Each score represents the average response for that duration. Pearson correlation coefficients and R2 values for each subject are also displayed. (0.10 MB DOC) [file pone.0010324.s001.doc]

| **Supplementary Table 1** | | |  |  |  |  |  |  |
| --- | --- | --- | --- | --- | --- | --- | --- | --- |
| **Subject ID** | **2-sec** | **4-sec** | **6-sec** | **8-sec** | **10-sec** | **12-sec** | **Pearson *r*** | **R^2^** |
| **Estimation** | |  |  |  |  |  |  |  |
| Con 1 | 2.0 | 3.6 | 5.4 | 6.6 | 8.2 | 10.2 | 0.9982 | 0.9964 |
| Con 2 | 2.7 | 4.9 | 7.6 | 9.7 | 12.6 | 14.7 | 0.9992 | 0.9985 |
| Con 3 | 3.0 | 5.0 | 7.3 | 9.6 | 11.9 | 13.9 | 0.9997 | 0.9994 |
| Con 4 | 3.2 | 5.5 | 8.9 | 11.0 | 14.2 | 16.5 | 0.9985 | 0.9969 |
| Con 5 | 1.9 | 3.1 | 4.5 | 5.4 | 7.0 | 8.4 | 0.9980 | 0.9960 |
| Con 6 | 1.9 | 3.3 | 4.8 | 6.5 | 7.8 | 9.6 | 0.9993 | 0.9986 |
| Con 7 | 2.0 | 3.1 | 4.2 | 5.8 | 7.2 | 8.7 | 0.9976 | 0.9951 |
| Con 8 | 1.9 | 3.3 | 5.1 | 7.7 | 9.0 | 11.2 | 0.9966 | 0.9932 |
| Con 9 | 1.2 | 3.0 | 4.3 | 5.3 | 7.5 | 8.9 | 0.9957 | 0.9915 |
| Con 10 | 1.8 | 3.3 | 4.4 | 6.3 | 7.6 | 9.8 | 0.9957 | 0.9914 |
| Con 11 | 2.1 | 4.0 | 6.2 | 8.4 | 10.0 | 12.0 | 0.9991 | 0.9981 |
| Con 12 | 2.2 | 3.9 | 5.2 | 7.1 | 8.0 | 10.2 | 0.9963 | 0.9926 |
| Con 13 | 3.0 | 2.7 | 4.3 | 5.3 | 6.8 | 7.9 | 0.9739 | 0.9485 |
|  |  |  |  |  |  |  |  |  |
| XG | 2.6 | 4.7 | 7.4 | 10.0 | 12.3 | 14.6 | 0.9996 | 0.9992 |
| KQ-167 | 1.8 | 3.8 | 4.7 | 7.1 | 7.9 | 9.5 | 0.9924 | 0.9848 |
|  |  |  |  |  |  |  |  |  |
| **Production** | |  |  |  |  |  |  |  |
| Con 1 | 1420.6 | 3773.1 | 5000.0 | 6011.6 | 8015.6 | 9520.9 | 0.9940 | 0.9880 |
| Con 2 | 1006.1 | 2492.5 | 3906.9 | 5075.7 | 6236.2 | 7677.6 | 0.9990 | 0.9980 |
| Con 3 | 1774.0 | 3231.4 | 4755.3 | 6267.0 | 7736.1 | 9816.5 | 0.9982 | 0.9964 |
| Con 4 | 1588.2 | 3407.2 | 4960.0 | 6502.6 | 8274.6 | 9904.1 | 0.9997 | 0.9995 |
| Con 5 | 981.0 | 3058.3 | 5377.9 | 7503.9 | 9500.6 | 11320.6 | 0.9993 | 0.9986 |
| Con 6 | 2697.4 | 4802.2 | 7164.0 | 9262.0 | 11904.6 | 13956.2 | 0.9995 | 0.9991 |
| Con 7 | 2504.8 | 5474.0 | 7974.6 | 10690.9 | 13978.0 | 16642.4 | 0.9994 | 0.9988 |
| Con 8 | 2033.2 | 3424.3 | 5281.5 | 6270.2 | 7704.2 | 9372.1 | 0.9978 | 0.9957 |
| Con 9 | 3115.5 | 5975.0 | 8939.7 | 12251.6 | 15078.8 | 18405.4 | 0.9997 | 0.9994 |
| Con 10 | 1113.2 | 2902.5 | 4917.0 | 6698.1 | 8490.7 | 10079.8 | 0.9995 | 0.9989 |
| Con 11 | 2368.7 | 3498.1 | 4905.4 | 6583.0 | 7834.4 | 9213.0 | 0.9989 | 0.9978 |
| Con 12 | 1713.8 | 4404.1 | 5948.0 | 8112.3 | 10116.0 | 11802.9 | 0.9978 | 0.9956 |
| Con 13 | 2144.1 | 5031.2 | 7661.5 | 10420.0 | 13503.1 | 15973.6 | 0.9997 | 0.9994 |
|  |  |  |  |  |  |  |  |  |
| XG | 2056.1 | 3817.3 | 5198.8 | 6370.7 | 7379.0 | 8646.7 | 0.9956 | 0.9913 |
| KQ-167 | 1366.2 | 3513.8 | 6040.6 | 7398.8 | 8877.7 | 11232.3 | 0.9955 | 0.9910 |
|  |  |  |  |  |  |  |  |  |
| **Reproduction** | |  |  |  |  |  |  |  |
| Con 1 | 1937.2 | 4881.8 | 7071.2 | 8285.8 | 10136.0 | 12136.0 | 0.9926 | 0.9852 |
| Con 2 | 2234.2 | 4033.6 | 6095.4 | 7326.0 | 9811.0 | 11803.6 | 0.9974 | 0.9948 |
| Con 3 | 2123.4 | 3753.2 | 5309.8 | 7405.8 | 9110.6 | 10590.2 | 0.9990 | 0.9980 |
| Con 4 | 2009.8 | 4044.0 | 5606.2 | 7625.2 | 9884.8 | 11709.8 | 0.9990 | 0.9980 |
| Con 5 | 1973.6 | 3866.8 | 5189.0 | 7325.8 | 8393.2 | 10520.4 | 0.9973 | 0.9946 |
| Con 6 | 2369.8 | 4793.4 | 6006.2 | 8039.6 | 9971.4 | 12406.4 | 0.9966 | 0.9933 |
| Con 7 | 1793.0 | 4088.0 | 5869.4 | 7588.8 | 9545.2 | 11729.6 | 0.9990 | 0.9981 |
| Con 8 | 1713.4 | 4316.2 | 6477.2 | 7756.0 | 9985.8 | 11299.6 | 0.9943 | 0.9886 |
| Con 9 | 1963.6 | 3027.2 | 4756.0 | 5892.4 | 9020.6 | 9137.6 | 0.9814 | 0.9631 |
| Con 10 | 1552.6 | 3490.8 | 5761.0 | 7471.2 | 9393.0 | 11408.0 | 0.9995 | 0.9990 |
| Con 11 | 2093.2 | 4075.8 | 5787.2 | 7973.2 | 9139.6 | 10804.4 | 0.9972 | 0.9943 |
| Con 12 | 1811.6 | 3360.6 | 6207.4 | 7482.0 | 9995.6 | 12013.4 | 0.9968 | 0.9937 |
| Con 13 | 1628.8 | 3203.4 | 5447.4 | 7168.0 | 9147.8 | 10996.0 | 0.9994 | 0.9988 |
|  |  |  |  |  |  |  |  |  |
| XG | 2735.5 | 4702.0 | 6071.6 | 7821.7 | 8841.6 | 11271.5 | 0.9955 | 0.9910 |
| KQ-167 | 2178.3 | 3912.5 | 5627.4 | 7266.7 | 8663.3 | 10926.8 | 0.9984 | 0.9968 |
